# Dietary total antioxidant capacity is inversely related to central adiposity as well as to metabolic and oxidative stress markers in healthy young adults

**DOI:** 10.1186/1743-7075-8-59

**Published:** 2011-08-22

**Authors:** Helen Hermana M Hermsdorff, Blanca Puchau, Ana Carolina P Volp, Kiriaque BF Barbosa, Josefina Bressan, M Ángeles Zulet, J Alfredo Martínez

**Affiliations:** 1 Department of Nutrition, Food Science, Physiology and Toxicology, University of Navarra. Pamplona, Spain; 2 Department of Clinical and Social Nutrition, Federal University of Ouro Preto, Ouro Preto, Brazil; 3 Nutrition Center, Federal University of Sergipe, Aracaju, Brazil; 4 Department of Nutrition and Health, Federal University of Viçosa. Viçosa, Brazil

**Keywords:** Antioxidants, Central obesity, ox-LDL, Oxidative stress, Arteriosclerosis

## Abstract

**Background:**

Dietary total antioxidant capacity (TAC) has been assumed as a useful tool to assess the relationship between the cumulative antioxidant food capacity and several chronic disorders. The aim of this cross-sectional study was to investigate the potential relationships of dietary TAC with adiposity, metabolic and oxidative stress markers in healthy young adults.

**Methods:**

This study enrolled 266 healthy subjects (105 men/ 161 women; 22 ± 3 years-old; 22.0 ± 2.7 kg/m^2^). Dietary intake, anthropometry, blood pressure, lifestyle features, and biochemical data were assessed with validated procedures.

**Results:**

In linear regression analyses, dietary TAC values were inversely associated with glycemia, total cholesterol:HDL-c ratio, triglycerides and oxidized-LDL concentrations, and positively associated with HDL-c concentrations, independently of gender, age, smoking status, physical activity, vitamin use supplement, waist circumference, energy intake, fatty acid intake. In addition, plasma TAC was negatively correlated with ox-LDL concentrations (*r*= -0.20, *P *= 0.003), independently of the assessed confounding variables. Finally, dietary TAC values were inversely related to waist circumference values (*r*= -0.17, *P *= 0.005) as well as to lower mild central obesity occurrence (waist circumference ≥ 80/ 94 cm for women/ men, respectively).

**Conclusion:**

Dietary TAC values are inversely associated with glucose and lipid biomarkers as well as with central adiposity measurements in healthy young adults, indicating dietary TAC as a useful tool to assess the health benefits of cumulative antioxidant capacity from food intake. In addition, the independent and inverse relationships of ox-LDL concentrations with dietary and plasma TAC respectively suggest a putative role of antioxidant rich-diet in the link between redox state and atherogenesis at early stage.

## Background

Dyslipidemias and insulin resistance constitute major risk factors of cardiovascular diseases (CVD) and related-features [1]. Furthermore, oxidative stress impairment or altered antioxidant status have been suggested as pivotal keys in the onset of certain chronic diseases such as metabolic syndrome (MS), type 2 diabetes and CVD [2,3]. In this sense, oxidized low-density lipoprotein (ox-LDL), a recognized oxidative stress marker, has been positively associated with central obesity [4], metabolic syndrome manifestations [5] and subclinical atherosclerosis [6].

In turn, dietary total antioxidant capacity (TAC) has been assessed in order to altogether capture synergic antioxidant/redox activities of single antioxidant compounds from diet [7]. Despite some authors have debated about the applicability of the extrapolation of dietary TAC data to its antioxidant contribution *in vivo *[8,9], this dietary index has been a relevant tool in epidemiological studies [10,11]. In this sense, increased dietary TAC has been associated with higher diet quality scores [12] as well as with improved values concerning glucose metabolism [13] and inflammatory status [14,15] in middle-aged people. In addition, dietary TAC has been recently associated with a lower risk for ischemic stroke in Italian cohort [16]. However, the relationship of dietary TAC with biomarkers has been only modestly investigated in young adult people [17,18], which is of great interest to provide new light for early an association of overall antioxidant intake with metabolic and oxidative biomarkers *in vivo*. Indeed, the association between this dietary TAC and ox-LDL concentrations has not been apparently reported.

Overall, the present study assessed the potential association of dietary TAC with adiposity as well as with metabolic and oxidative stress markers in healthy young adults with emphasis on plasma ox-LDL concentrations, as a relevant oxidative stress marker and an atherosclerosis predictor.

## Subjects and Methods

### Subjects

Participants of the current research were involved in a study of Interuniversity Cooperation between the Federal University of Viçosa (Brazil, CAPES-MECD-DGU 109/06) and the University of Navarra (Spain, PHB-2005-0119-PC). Thus, a group of 266 subjects from Brazil (57 men and 66 women) and Spain (48 men and 95 women), with a mean age of 22 ± 3 years-old (range: 18-35 years) and a mean body mass index (BMI) of 22.0 ± 2.7 (range: 18.5-34.9 kg/m^2^), were enrolled in this study.

The volunteers were recruited through magazines, radio, web page, and intranet tools at both Universities. In the enrollement message, the age range (18-35 years old) was mentioned as well as relevant clinical information for those interested in participating in this cross-sectional nutritional study. Exclusion criteria were any diagnosed organic underlying disease (gastrointestinal, kidney, liver, respiratory or heart disease), cancer, infectious and inflammatory disorders, diabetes (fasting glucose level > 126 mg/dl), hypertension (systolic and diastolic blood pressure values ≥ 140 and 90 mmHg, respectively), pregnancy, disorders affecting body composition (e.g. lipodystrophy and Cushing syndrome) or blood lipid-lowering treatments. Other exclusion criteria were recent follow up of diets designed for weight loss or unstable weight in the past 3 months. The present study was conducted according to the guidelines laid down in the Declaration of Helsinki and all procedures involving human subjects/patients were approved by the appropriate human research review boards at each location: Ethics Committee in Human Research of the Federal University of Viçosa (ref. n° 009/2006) and Investigation Ethics Committee of the Clínica Universidad de Navarra (ref. n° 79/2005). Written informed consent was obtained from all the subjects/patients.

### Dietary intake assessment

In the Brazilian sample, dietary intake information was obtained by a 3 day-record. Daily food consumption was estimated as a 3d-mean of portion size for each consumed food item, considering in addition to preparation (crude or cooked), and edible portions. Nutrient intake was estimated using the Diet Pro 5i^® ^software (AS Sistemas, Viçosa, Brazil), adapted with the latest available information from the food composition tables for Brazil [19,20]. In the Spanish sample, dietary intake information was obtained by a semi-quantitative food frequency questionnaire with 136 food-items, which is validated for Spanish people [21,22]. Daily food consumption was estimated as frequency × portion size for each consumed food item. Nutrient intake was estimated using an *ad hoc *computer program specifically developed for this aim, which displays the latest available information included in the food composition tables for Spain [23,24].

Furthermore, dietary TAC from dietary intake information, expressed as mmol (of Trolox equivalent)/d, was calculated by a proxy estimation previously validated to the 3 day-record as well as for the food-frequency questionnaire [18].

### Clinical and biochemical assessments

Anthropometric determinations were taken using standard measurement procedures, in accordance to previously described protocols [25] as agreed by both universities participating in the study. Thus, BMI was calculated by the ratio between weight (kg) and the squared height (m^2^), which was applied to categorize normal-weight (18.5-24.9 kg/m^2^), overweight (25-29.9 kg/m^2^), and obese (BMI ≥ 30 kg/m^2^) subjects, according to the World Health Organization criteria [26]. Waist circumference was used as a central adiposity indicator, considering values higher than 80 and 94 cm for women and men, respectively, as an indicator of mild central obesity. Systolic and diastolic blood pressures were measured following World Health Organization guidelines [27].

Venous blood samples were drawn after a 12 h overnight fast by venipuncture. The EDTA-plasma and serum samples were separated from whole blood by centrifugation (2,205 *g *× 15 min at 4°C) and were frozen immediately at -80°C until assay. Serum concentrations of triglycerides, total cholesterol (TC), high density lipoprotein-cholesterol (HDL-c), glucose and insulin were measured by standard methods as previously described [17,25]. Serum low-density lipoprotein-cholesterol (LDL-c) and insulin resistance as HOMA-IR were calculated as described by Friedewald et al. [28] and Matthews et al. [29] equations, respectively. TC:HDL-c and LDL-c:HDL-c ratios also were calculated, since they are independent predictors of the risk for CVD [30,31]. Hypercholesterolaemia was considered as a total cholesterol concentration ≥ 200 mg/dl [1]. Plasma ox-LDL was measured using ELISA kits from Mercodia (Uppsala, Sweden), based on the mouse monoclonal antibody 4E6, which is directed against a conformational epitope in oxidized ApoB-100 [32,33]. Finally, plasma TAC was determined using a commercial colorimetric kit (Cayman Chemical Corporation, Ann Arbor, USA), based on the inhibition the oxidation of ABTS^® ^(2,2-Azino-di-[3-ethylbenzthiazoline sulphonate]) to ABTS^®·+^, which is subsequently quantified as mmol Trolox equivalent [34,35].

### Other variable assessment

For lifestyle variables, the participants were asked about their smoking status (never, former, or current smokers) and about vitamin supplement use (Yes/No). With respect to physical activity, the participants declared whether they took regular physical activity (Yes/No), and if so, the type and the volume of activity (h/week). To quantify the volume of activity, a metabolic equivalent (MET) index was also computed by assigning a multiple of resting metabolic rate (MET score) to each activity [36], followed by the sum over all activities to obtain a value of overall weekly MET/h as described elsewhere [37].

### Statistical analysis

Results are shown as mean ± standard deviations (SD) or median (interquartile interval), depending on the variable distribution as determined by the Shapiro-Wilk test. Non-normally distributed variables were log-transformed before statistical analyses. Dietary intakes were adjusted for the daily energy intake, while biochemical variables, when considered outcomes, were adjusted for study center, both by the residuals method, applying separate models among women and men [38]. To assess the associations of dietary TAC values with anthropometric, clinical and lifestyle characteristics of the participants, we categorized the participants by tertiles of this specific dietary index. Linear trends were assessed by assigning the median value to each tertile of dietary TAC and modeling these values as a continuous variable. Comparisons between three groups were performed by chi-square tests (categorical variables) or by one-factor ANOVA tests (continuous variables), while the *post hoc *Bonferroni test was used to correct the impact of multiple comparisons.

Moreover, β-coefficients and 95% confidence intervals (CIs) were calculated in multivariate linear regression models to assess the association of dietary TAC values (independent continuous variable) with the investigated glucose and lipid biomarkers (dependent variables). Linear regression models were controlled by gender, age (years), waist circumference (cm), daily energy intake (kcal/d), physical activity during leisure time (METs-hour per week), smoking status (never, former and current smokers), vitamin supplement use (Yes/ No), and monounsaturated (MUFA): saturated fatty acid (SFA) ratio intake, since these variables were considered potential confounding factors. In addition, partial correlations were established to evaluate the potential relations between dietary TAC, plasma TAC and ox-LDL concentrations as well as potential links between dietary TAC and waist circumference as a central adiposity indicator.

Furthermore, we used stepwise multiple regressions [38] to identify the variability impact of the consumed food-items concerning dietary TAC values of the participants of this study. Statistical analyses were performed with SPSS 15.0 software (SPSS Inc., Chicago, IL, USA) for Windows XP (Microsoft, USA). A *P*-value < 0.05 was considered as statistically significant.

## Results

**Figure 1:**
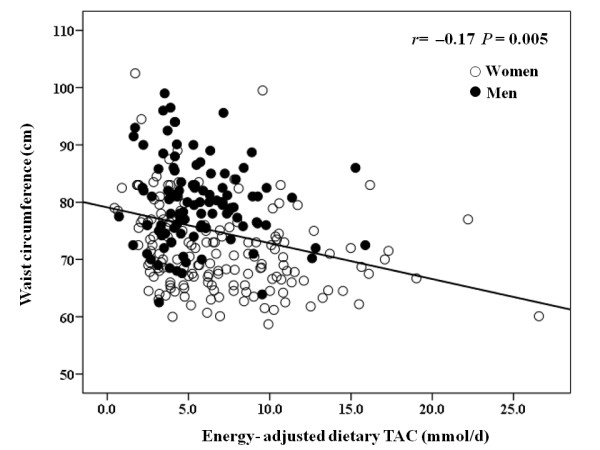
**Association between dietary TAC and waist circumference values**. *P*-value from partial correlation, adjusted for gender (n = 266).

Anthropometric, clinical and lifestyle characteristics were examined by tertiles of dietary TAC values (Table 1). Thus, those subjects included in the highest tertile were older subjects, had lower values for diastolic blood pressure and higher values for physical activity counts (METs), while reported higher vitamin supplement use as compared with those of the lowest tertile (*P *< 0.05). In addition, the participants included in the last tertile of dietary TAC consumption had significantly lower waist circumference values as well as lower occurrence of mild central obesity (as waist circumference ≥ 80 and 94 cm for women and men, respectively). Indeed, dietary TAC values were negatively and significantly correlated (*r *= -0.17, *P *< 0.05) with waist circumference values in the partial correlation adjusted for gender (Figure 1).

**Table 1:** Anthropometric, clinical, and lifestyle characteristics, according to tertiles (T) of energy-adjusted dietary TAC (n = 266)

	Energy-adj. dietary TAC (mmol/d)			
	T1 (< 1.6)	T2 (1.6-5.9)	T3 (≥ 5.9)	*P*-value^a^
Participants (n)	86	90	90	
Men (n, %)	34 (39.5)^b^	35 (38.8)	34 (37.8)	0.994
Age (y)	21 ± 3^e^	22 ± 3	23 ± 4	*0.031*
Body mass index (kg/m^2^)	21.8 ± 3.1	22.5 ± 2.6	21.9 ± 2.4	0.201
Waist circumference (cm)	76.1 ± 9.0^e^	75.5 ± 8.1	72.9 ± 7.6	*0.027*
Overweight/ Obesity (n, %)^c^	11 (33.3)	13 (39.5)	9 (27.3)	0.731
Mild central obesity (n, %)^d^	12 (14.0)	12 (13.3)	2 (2.2)	*0.014*
Physical activity practice (n, %)	47 (54.6)	54 (60.0)	60 (66.7)	0.087
METs (h/ week)	32 (18;59)^e^	40 (20; 85)	98 (43;186)	*0.037*
Former/ Current smoker (n, %)	14 (16.9)	24 (26.7)	26 (28.9)	0.106
Vitamin supplement use (n, %)	10 (11.6)	23 (25.5)	28 (31.1)	*0.031*
Hypercholesterolaemia (n, %)	13 (39.4)	8 (24.2)	12 (36.4)	0.132
Systolic BP (mmHg)	110 ± 9	113 ± 11	112 ± 10	0.081
Diastolic BP (mmHg)	72 ± 8^e^	69 ± 9	66 ± 8	*< 0.001*

^a^*P*-value from one-factor ANOVA test or χ^2 ^test, for continuous or categorical variables, respectively. Non-normally distributed variables were log-transformation before analyses.

^b^Continuous variables are presented as mean ± SD or median (interquartile interval), while categorical variables are presented as number of the participants (percentages).

^c^Overweight/Obesity occurrence, according to BMI values (≥ 25 and ≥ 30 kg/ m^2^, respectively).

^d^Mild central obesity occurrence, according to waist circumference values (≥ 80 and ≥ 94 cm for women and men, respectively).

^e^*P *< 0.05, T1 vs. T3; from the *post hoc *Bonferroni test for multiple comparisons.

TAC, total antioxidant capacity; MET, metabolic equivalent index; BP, blood pressure.

Moreover, those participants who were included in the third tertile of dietary TAC presented higher consumption of olive oil, fruits, vegetables, fruit juices, fish, coffee and red wine as well as higher intake values of protein, lipids, monounsaturated fatty acid (MUFA), and dietary fiber, while lower intake values for saturated fatty acid (SFA) were found as compared with subjects in the lowest tertile (Table 2). In addition, the food-groups consumed by the participants with higher contribution to dietary TAC values were vegetables, fruits and fruit juices (*R*^2 ^= 0.69). Other important contributing food items, such as olive oil, coffee, fish, and legumes, explained altogether 22% of total variability in dietary TAC. Fruits and vegetables remained as the highest contributors to dietary TAC when the analysis was performed by study center (data not shown).

**Table 2:** Food and nutrients consumption, according to tertiles (T) of energy-adjusted dietary TAC (n = 266)

	Energy-adj. dietary TAC (mmol/d)			
	T1 (< 1.6)	T2 (1.6-5.9)	T3 (≥ 5.9)	*P*-value^a^
Participants (n)	86	90	90	
Energy intake (kcal)	2758 ± 699	2540 ± 922	2785 ± 855	0.108
Carbohydrate (% EI)	50.5 ± 7.9^b,c^	47.3 ± 8.4 ^d^	44.5 ± 8.0	*< 0.001*
Protein (%EI)	15.0 ± 2.5 ^b,c^	16.5 ± 2.7 ^d^	17.5 ± 2.6	*< 0.001*
Lipids (%EI)	33.3 ± 6.0 ^b,c^	36.1 ± 6.1	36.9 ± 6.0	*0.002*
MUFA (%EI)	6.3 ± 4.2^b,c^	12.4 ± 6.2^d^	15.7 ± 4.5	*0.001*
PUFA (%EI)	4.6 ± 2.1^c^	5.5 ± 2.7	5.4 ± 1.9	*0.017*
SFA (%EI)	12.2 ± 3.6 ^b,c^	10.6 ± 4.0^d^	9.1 ± 3.8	*0.003*
Dietary fiber (g/d)	24.2 ± 13.2 ^b^	25.3 ± 15.0	29.6 ± 12.7	*0.023*
Olive oil (ml/d)	4 ± 16 ^b,c^	19 ± 27 ^d^	39 ± 33	*< 0.001*
Fruits (g/d)	154 ± 120 ^b,c^	245 ± 190^d^	393 ± 300	*< 0.001*
Vegetables (g/d)	94 ± 68 ^b,c^	253 ± 211^d^	572 ± 395	*< 0.001*
Fruit juice (ml/d)	67 ± 95^b^	91 ± 122	169 ± 233	*< 0.001*
Cereals (g/d)	145 ± 114	167 ± 104	177 ± 83	0.103
Legumes (g/d)	29 ± 20	28 ± 31	20 ± 13	0.127
Red meats (g/d)	99 ± 67	96 ± 54	84 ± 61	0.214
Fish (g/d)	16 ± 43 ^b,c^	41 ± 52 ^d^	93 ± 63	*< 0.001*
Nuts (g/d)	8 ± 10	7 ± 8	15 ± 32	0.281
Coffee (ml/d)	38 ± 63^b^	59 ± 82	68 ± 84	*0.028*
Beer (ml/d)	31 ± 62	31 ± 49	58 ± 107	0.069
Red wine (ml/d)	6 ± 5^b^	7 ± 4^d^	50 ± 18	*0.009*

^a^*P*-value from one-factor ANOVA test.

^b^*P *< 0.05, T1 vs. T3; ^c^*P *< 0.05, T1 vs. T2; ^d^*P *< 0.05, T2 vs. T3, from the *post hoc *Bonferroni test for multiple comparisons.

dietary TAC, dietary total antioxidant capacity; EI, energy intake; MUFA, monounsaturated fatty acid; PUFA, polyunsaturated fatty acid; SFA, saturated fatty acid.

Regarding the association between dietary TAC and glucose profile, dietary TAC values were inversely associated with glycemia and HOMA-IR (*P *< 0.05), independently of gender, age, waist circumference, smoking habit, physical activity counts, vitamin supplement use (Table 3). Furthermore, higher dietary TAC values were statistically associated with lower values to TC concentrations, TC:HDL-c ratio, ox-LDL and triglycerides concentrations as well as with higher values to HDL-c concentrations, independently of the same covariates (Table 3). Interestingly, similar outcomes are found when waist circumference (cm) was substituted by BMI (kg/m^2^) or by mild central obesity occurrence, as categorical covariate (data not shown).

**Table 3:** Association of dietary TAC values (as independent variable) and glucose and lipid profile (as dependent variables) in the participants of the study (n = 266)

Dependent variables^a^	Energy-adj. dietary TAC (mmol/d) as independent variable	
	
	Model 1^b^	Model 2^b^
Glucose (mg/dl)	**-0.002 (-0.003; -0.001)**^c^	**-0.002 (-0.003;-0.001)**
Insulin (μIU/l)	**-0.009 (-0.0180; -0.001)**	-0.006 (-0.016; 0.003)
HOMA-IR	**-0.011 (-0.021; -0.002)**	-0.008 (-0.019; 0.004)
TC (mg/dl)	**0.003 (0.001; 0.006)**	0.002 (-0.002; 0.005)
HDL-c (mg/dl)	**0.007 (0.004; 0.010)**	**0.005 (0.001; 0.008)**
LDL-c (mg/dl)	0.004 (-0.001;0.008)	-0.001 (-0.003; 0.005)
TC:HDL-c ratio	**-0.004 (-0.007; -0.001)**	**-0.003 (-0.006;-0.001)**
LDL-c:HDL-c ratio	-0.003 (-0.008; 0.001)	-0.004 (-0.008; 0.001)
ox-LDL (U/l)^d^	**-2.772 (-3.226; -2.318)**	**-1.976 (-2.422; -1.531)**
TG (mg/dl)	**-0.013 (-0.020; -0.007)**	**-0.007 (-0.013; -0.001)**

^a^Non-normally distributed variables were log-transformed before regression analyses and, adjusted for study center by residual method.

^b^Model 1: multivariate linear regression adjusted for gender, age (years), waist circumference (cm), daily energy intake (kcal/d), smoking habit (never or smoker/former), METs (h/week), and vitamin supplement use (Yes/ No). Model 2: multivariate linear regression adjusted for as model 1 plus MUFA: SFA ratio intake.

^c^Data are β-coefficient (95% Confidence Interval). Bold style to significant associations.

^d^n = 224, for this variable.

TAC, total antioxidant capacity; HOMA-IR, insulin resistance index; TC, total cholesterol; HDL-c, high density lipoprotein-cholesterol; LDL-c, low density lipoprotein-cholesterol; ox-LDL, oxidized low density protein cholesterol; TG, triglycerides.

Since dietary fatty acids have been related to atherogenesis and CVD, linear regression models were also adjusted for MUFA:SFA ratio. In this case, the associations of dietary TAC values with glycemia, TC:HDL-c ratio, HDL-c, ox-LDL and triglycerides concentrations maintained the trend and the statistical significance, while its associations with insulin, HOMA-IR, and TC lost the statistical significance (Table 3). For this reason, the interaction between dietary TAC and MUFA:SFA ratio was tested, but any statistical interplay was found (*P *> 0.05).

**Figure 2:**
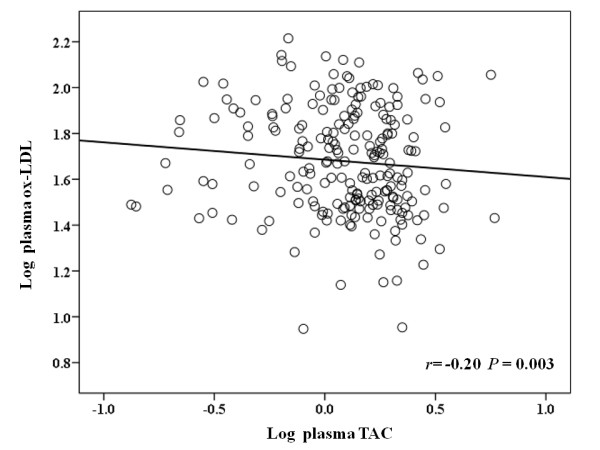
**Association between log plasma TAC and log ox-LDL**. *P*-value from partial correlation, adjusted for study center, gender, age, energy intake, waist circumference and LDL-c concentrations (n = 224).

Finally, dietary TAC values were positively related with plasma TAC concentrations in the participants of the study, although statistical significance was not identified (*r *= 0.11, *P *= 0.131) in the partial correlation, adjusted for study center, gender, age, and energy intake. Interestingly, plasma TAC was inversely correlated with ox-LDL concentrations (*r*= -0.20, *P *= 0.003), independently of the study center, gender, age, energy intake, waist circumference and LDL-c concentrations (Figure 2).

## Discussion

The first relevant finding of this study was the lower occurrence of mild central obesity in those individuals included in the last tertile of dietary TAC, while dietary TAC values were inversely correlated to waist circumference values in a young population. Other studies have reported an inverse association of the dietary TAC and dietary antioxidants with obesity indicators, while abdominal obesity has been associated with decreased serum antioxidants concentrations in other age groups [10,39-41]. Thus, in the line with existing literature, data from our study indicate a relationship between dietary antioxidant consumption, body fat distribution and antioxidant status in healthy young people.

In this study, the dietary TAC was also inversely associated with glucose biomarkers. This finding is in agreement with other studies that have reported an inverse relationship between dietary TAC and glucose biomarkers in young and middle-aged subjects [13,18], reinforcing the hypothesis of an interactive influence between oxidative stress, pro-inflammation and insulin resistance [2,42].

In turn, the association of a higher dietary TAC with lower values of some specific lipid biomarkers appears to be reported apparently for the first time in the present study. In fact, an increased consumption of antioxidant-rich foods, such as fruits, vegetables, olive oil, nuts, red wine, seafood and legumes, has resulted in an improvement in the lipid profile, with increased HDL-c and decreased LDL-c and triglycerides concentrations in some intervention trial studies [43-46]. Likewise, polyphenols and carotenoids have the ability to reduce cholesterol absorption, to increase cholesterol and fecal bile excretion, to inhibit cholesterol synthesis and to stimulate the expression and activity of the LDL receptors [47]. Since these compounds might contribute to dietary TAC values, they could be related to potential hypocholesterolemic mechanisms involving the dietary healthy index. In this context, our findings suggest that dietary TAC is a reliable indicator to assess the relationship of antioxidant-food items altogether with glucose and lipid biomarkers, despite other studies could contribute to establish molecular and cellular mechanisms as well as its potential application in the treatment of chronic disorders *in vivo*.

Other relevant outcome of this cross-sectional study, which is also apparently reported for the first time, was the inverse and independent association of dietary TAC values with ox-LDL, a recognized oxidative stress marker and independent risk factor for MS and CVD [5,6]. In fact, a high consumption of antioxidant-rich foods might decrease oxidation in the low-density lipoprotein. On one hand, by scavenging free radicals and by sparing lipophilic antioxidant content of lipoproteins and, on the other hand by increasing the plasma TAC availability [47-50]. In this sense, the independent correlation between plasma TAC and ox-LDL concentrations observed in this study might explain, at least in part, the potential effect of dietary TAC on this oxidative stress marker. In fact, lipid-soluble antioxidants (e.g. carotenoids) are carried in LDL; therefore, an increase in the antioxidant-substrate could be reflected in higher LDL resistance to oxidation [51].

Moreover, we had previously reported an inverse association between dietary TAC values, plasma C-reactive protein and gene expression of the nuclear factor-kappa-B, interleukin-1 receptor-1, interleukin-6 and tumor necrosis factor-alpha [17]. Since ox-LDL is able to induce a pro-inflammatory status by the activation of the nuclear factor-kappa-B, a redox-sensitive and pro-inflammatory transcriptional factor [42,52], our previous and current findings suggest jointly a putative role of antioxidant rich-foods, expressed by dietary TAC values, in the link between oxidative stress and inflammation in which TAC and ox-LDL are likely interacting.

In turn, when MUFA:SFA ratio was included in the linear regression models, the prediction power of dietary TAC on glucose and lipid biomarkers was attenuated or lost in some cases (HOMA-IR, TC and triglycerides). This finding suggests potential synergistic actions of the subtype of fat intake and dietary antioxidant content in the glucose and lipid metabolism, since the replacement of SFA to MUFA resulted in an improvement in the lipid profile and glycemic control in other intervention trials [43,53-55]. Likewise, since dietary TAC values were inversely correlated to waist circumference values in this study and, body adiposity has been positively associated with pro-inflammatory and oxidative stress markers [4,10,56,57], the inverse relationships of dietary TAC values with some of the investigated biomarkers could be biased by a lower central obesity among those participants with higher dietary TAC values. However, the associations between dietary TAC and the studied markers maintained the trend and the statistical significance regardless of the waist circumference, suggesting that the effects of dietary TAC on glucose and lipid profile in this study were independently from body fat distribution. In addition, our sample presented higher number of women (60%). The hormone estrogen (17β-estradiol in particular) has been noted to have antioxidant and antilipidemic properties [58], which might influence in the association of dietary intake with the studied biomarkers, depending on menstrual cycle status. However, we adjusted dietary total antioxidant by energy in separated models for women and men and we assessed the association of dietary TAC values with the investigated glucose and lipid biomarkers in multivariate linear regression models controlled by gender. Thus, our main study outcomes should be independent from gender and sex-hormonal effect. Moreover, estradiol concentrations have not been able to modify the oxidative and inflammation status in young women, regardless menstrual cycle phase [58].

Our study had certain limitations. First, since the nature of this study is cross-sectional, we cannot prove that the reported associations are causal, although we controlled for several potential covariates. Second, the use of different dietary assessment methods (food-frequency questionnaire and 3 day-record) by the study centers could provide differences in the information concerning dietary intake. However, both dietary assessment methods were validated to assess dietary TAC with a strong correlation between them [18]. At the same time, both dietary questionnaires have been successfully used to assess the relationship of dietary TAC values from habitual diet with biomarkers [13,14,17,18]. In addition, major outcomes of this study maintained the statistical significance after adjusting for study center. Finally, although the sample size is adequate from the standpoint of an initial association discovery, further replication in independent and larger samples would be convenient for a future translational application at a population level.

## Conclusion

In this cross-sectional study, dietary TAC values are inversely associated with glucose and lipid biomarkers as well as with central adiposity in healthy young adults, indicating dietary TAC as a useful epidemiological tool to assess to health benefits of a cumulative antioxidant capacity from food intake. In addition, the independent and inverse relationships of ox-LDL concentrations with dietary and plasma TAC suggest a putative role of an antioxidant rich-diet in the link between redox state and atherogenesis at early stage.

## Abbreviations

BMI: body mass index; CVD: cardiovascular disease; HDL-c: high density lipoprotein-cholesterol; MET: metabolic equivalent index; MUFA: monounsaturated fatty acid; MS: metabolic syndrome; LDL-c: low density lipoprotein-cholesterol; ox-LDL: oxidized low density lipoprotein; SFA: saturated fatty acid; TAC: total antioxidant capacity; TC: total cholesterol.

## Competing interests

The authors declare that they have no competing interests.

## Authors' contributions

HHMH: Design, field work, data collection, analysis, and writing of the manuscript. BP, ACPV, and KBFB: Design, field work, and data collection. JB: project leader in Brazil, design, financial management. MZ: project co-leader in Spain, design, financial management. JAM: project leader in Spain, general coordination, design, financial management, data interpretation. All authors assisted in editing the manuscript as well as they read and approved the final manuscript.
